# Actual and Illusory Perception in Parkinson's Disease and Dystonia: A Narrative Review

**DOI:** 10.3389/fneur.2018.00584

**Published:** 2018-07-20

**Authors:** Laura Avanzino, Mirta Fiorio, Antonella Conte

**Affiliations:** ^1^Section of Human Physiology, Department of Experimental Medicine, University of Genoa, Genoa, Italy; ^2^Department of Neurosciences, Biomedicine and Movement Sciences, University of Verona, Verona, Italy; ^3^Department of Human Neurosciences, Sapienza University of Rome, Rome, Italy; ^4^IRCCS Neuromed, Pozzilli, Italy

**Keywords:** Parkinson's disease, dystonia, temporal processing of sensory information, bodily illusion, proprioception

## Abstract

Sensory information is continuously processed so as to allow behavior to be adjusted according to environmental changes. Before sensory information reaches the cortex, a number of subcortical neural structures select the relevant information to send to be consciously processed. In recent decades, several studies have shown that the pathophysiological mechanisms underlying movement disorders such as Parkinson's disease (PD) and dystonia involve sensory processing abnormalities related to proprioceptive and tactile information. These abnormalities emerge from psychophysical testing, mainly temporal discrimination, as well as from experimental paradigms based on bodily illusions. Although the link between proprioception and movement may be unequivocal, how temporal tactile information abnormalities and bodily illusions relate to motor disturbances in PD and dystonia is still a matter of debate. This review considers the role of altered sensory processing in the pathophysiology of movement disorders, focusing on how sensory alteration patterns differ between PD and dystonia. We also discuss the evidence available and the potential for developing new therapeutic strategies based on the manipulation of multi-sensory information and bodily illusions in patients with these movement disorders.

## Introduction

A large body of evidence shows that altered sensory processing intervenes in the pathophysiology of movement disorders, including Parkinson's disease (PD) [for a review see (1)] and dystonia [for a review see (2)]. Sensory abnormalities in movement disorders have been reported by investigating proprioceptive (3–5) as well as tactile information processing (6–8) with various neurophysiological techniques. Some evidence on altered sensory processing also comes from experimental paradigms using bodily illusions (9, 10). The fact that sensory abnormalities are consistently present in patients which clinically manifest motor disturbances has raised the question whether sensory alterations participate in the pathophysiological mechanisms of motor disturbances in PD and dystonia through defective sensorimotor integration. It is still unknown whether the pattern of proprioceptive and tactile abnormalities and the pattern of bodily illusion alterations is similar in PD and dystonia.

In this narrative review, we examine the evidence on actual and illusory sensory perception in PD and dystonia and discuss the possible role of these abnormalities in the pathophysiology of movement disorders.

## Experimental approaches to investigate actual and illusory proprioceptive information in humans

Proprioception is the ability to sense the position and movements of our limbs and trunk independently of vision (kinaesthesia). The fundamental receptor involved in proprioception is the muscle spindle, which includes the primary and secondary endings of spindles. Primary endings, subserved by Ia afferents, respond to the size and speed of muscle length changes (11) and contribute to both the sense of limb position and movement (12). Secondary endings, subserved by group II afferents, only signal the length change and thus contribute to the sense of position (11). Once the signals from proprioceptors enter the spinal cord, the proprioceptive information is handled by a number of higher order neurons, distributed in the cerebellum and the cerebral cortex [for a review, see (13)]. The input to the cerebellum is mainly used for computations of predictive information (14), while that to the cerebral cortex is responsible for generating proprioceptive sensations (15, 16) and for controlling on-going actions (17).

Experimentally, it is possible to generate an artificial proprioceptive signal through the application of vibratory stimuli over the muscle (15, 16). Vibration of the muscle belly or tendon at 50–120 Hz produces a tonic vibration reflex, depending on the activation of muscle spindles and γ-motoneurons. Furthermore, when the vibrated arm is immobilized, the vibration stimulus evokes an illusion of movement corresponding to the activation of sensorimotor areas in the brain (18). Thus, the study of the tonic vibration reflex allows the integrity of the proprioceptive afferent pathways to be assessed, whereas movement illusion refers to the integration of the proprioceptive signal at a central level. The integration of proprioceptive inputs at a central level can also be tested by means of various tasks, which include position matching, testing the velocity discrimination threshold for detecting limb motion and testing the temporal discrimination threshold for distinguishing two successive movements (13). Lastly, it is worth noting that, during motor control, proprioception also serves as a means of building a correct internal model of movement, adopted for the feed-forward portion of motor control. Indeed, real-time movement control requires not only the ability to sense position and movements, but also to predict limb position through a correct internal model. The aim of feed-forward motor control is to create adequate anticipatory motor activity to achieve the desired performance. The cerebellum has been shown to be the key structure in feed-forward movement control (14, 19).

Somatosensory functions, especially proprioception have been investigated in humans even by exploiting experimental paradigms based on illusions. Although less is known about tactile and cross-modal illusions than about visual illusions, the former may offer interesting ways to understand brain mechanisms related to perceptions of both the body and of objects through the body. The proprioceptive function can be indirectly tackled by means of the rubber hand illusion (RHI) paradigm. The RHI, which is based on visual, tactile and proprioceptive inputs, sheds light on multisensory integration related to bodily awareness. This illusion was described for the first time by Botvinick and Cohen in 1998 and consists of the sense of ownership over an artificial hand (20). More precisely, when the participant's own hidden hand and an artificial visible hand are stroked synchronously (with two paintbrushes), the artificial hand is actually perceived as being the participant's own hand—subjective feeling of ownership—while the participant's hand is implicitly perceived as being located nearer to the artificial hand—proprioceptive drift (21). Similar asynchronous stroking does not evoke the illusion. The proprioceptive drift represents the recalibration of the perceived location of one's own hand toward the rubber hand and has been associated with the activity in a number of cortical and subcortical brain regions, such as the intraparietal cortex, the dorsal premotor cortex, the supplementary motor area, the cerebellum, the putamen, and the ventral thalamus (22, 23).

## Evidence on actual and illusory proprioceptive information processing in PD and dystonia

Strong evidence supports the notion that proprioceptive deficits in PD and dystonia are not peripheral in origin, but central. Indeed, tonic vibration reflex is normal in PD and dystonia (24, 25) (Table 1), thereby suggesting that proprioceptors normally convey information to the central nervous system. Accordingly, microneurographic recordings of muscle spindles in PD are normal (66).

**Table 1:** Sensory abnormalities in Parkinson's disease and Dystonia.

**Sensory function**	**Task**	**Parkinson's disease**	**Dystonia**	**References**
Proprioception	Muscle vibration	Normal TVR	Normal TVR, but abnormal arm movement perception	(24–27)
	Limb position matching	Abnormal	Abnormal	(4, 28–30)
	Limb motion discrimination threshold	Increased	Normal	(31, 32)
	Haptic acuity	Decreased	Decreased	(5, 33)
	Movement control	Dependence on vision for defective proprioception	Impaired reaching movements and feed-forward movement control	(2, 34–41)
Tactile	STDT	Increased but normal at disease onset	Increased in patients and in unaffected relatives of dystonic patients	(6, 7, 42–63)
Multimodal	RHI	Increased	Abnormal only in FHD	(9, 10)
	Aristotele illusion	Normal	Abnormal only in FHD	(64, 65)

In PD, abnormal processing of proprioceptive information is likely to occur at a subcortical/cortical level. Impaired kinaesthetic sensitivity to changes in limb position and limb motion at both distal and proximal arm joints has been observed in PD patients (4, 28) (Table 1). More recently, (5) also explored haptic acuity in PD patients by attaching robotic manipulators to the arm that exerts forces to create the illusion of contours around a simulated object. Haptic acuity and sensitivity were decreased in PD patients during both active and passive exploration (5), thus suggesting that PD affects the early stages of somatosensory integration, which in turn subsequently has an impact on sensorimotor integration. In accordance with this hypothesis, the temporal discrimination threshold for distinguishing two successive movements was increased in PD (31); moreover, PD patients made more errors than controls when they were asked to match the limb position to a visual illustration of the arm (29). A large body of evidence suggests that the dependence of PD patients on visual cues in tasks ranging from arm movements to responses to postural perturbation or walking may, as a consequence of defective somatosensory and sensorimotor integration, be due to defective proprioceptive function (34–38).

Boecke et al. (67) showed, by means of positron-emission tomography scans during a high-frequency vibratory stimulus, that the activation of contralateral primary and secondary somatosensory cortices, primary motor and lateral premotor cortices and basal ganglia is reduced while that of the ipsilateral sensory cortical areas is enhanced. This finding, together with the improvement observed in proprioceptive function after bilateral STN-DBS in PD (68, 69), suggests that altered connectivity between the basal ganglia and the somatosensory cortex may be responsible for abnormal proprioceptive processing in PD.

With regard to the pharmacological effects, there is evidence of no effect or of even a greater proprioceptive dysfunction after dopaminergic treatment administration in PD patients when proprioceptive information is used for motor control (sensorimotor integration process) (70–72). Indeed, the experimental paradigm that yielded negative results was based on arm position matching or reaching tasks. The negative effect of medication on motor performance has been attributed to the dyskinetic effect of dopaminergic therapy (70). However, the results of one study that did adopt a proprioceptive perceptual task without any motor component showed that perceptual sensitivity increased after dopamine replacement therapy (73). It has been speculated that improved availability of dopamine might improve the activity of basal ganglia neurons that respond to multimodal sensory stimulation, as has clearly emerged from animal parkinsonian models (74, 75).

In dystonia, there is evidence that points to both abnormalities in proprioceptive processing at a cortical level for sensorimotor integration processes and abnormalities in the internal model of movement that are likely to be caused by a defective integrating function of the cerebellum that prevents the creation of a correct internal model (2, 3). The perception of limb movement in dystonia is acknowledged to be abnormal, whereas results on the perception of limb position are contrasting. In this regard, patients with focal hand dystonia display a temporal discrimination threshold for distinguishing two successive movements that is comparable to that of controls (32), though they make more errors when they are required to compare the amplitudes of two consecutive movements (30). Furthermore, the perception of arm movement during tonic vibration reflex or during illusory movements induced by vibration in an immobilized arm has been found to be abnormal (26, 27) (Table 1). Interestingly, proprioceptive acuity has been found to be decreased even in non-dystonic muscles, e.g., in the limb muscles of patients affected by spasmodic dystonia (33). These sensory abnormalities may impair the process of sensorimotor integration in dystonia. Indeed, in the absence of visual information, patients with dystonia of the upper limb (76) and cervical dystonia (77) display impaired reaching movements with the upper limb toward a specific target, which points to a possible failure to integrate proprioceptive information with the motor output (78). A large body of evidence based on neurophysiological and neuroimaging techniques shows that the functional and anatomical correlates of impaired proprioceptive processing are located at the subcortical (basal ganglia) and cortical (sensorimotor cortex) levels [for a review see (2, 79)].

The role played by the cerebellum in integrating proprioceptive inputs appears to be particularly relevant to the pathophysiology of dystonia. We recently demonstrated that the internal forward model of a motor act is abnormal in dystonia by displaying that when subjects had to rely solely on this forward model to predict the temporal outcome of a motor act, they were unsuccessful (39, 40). For this purpose, we adopted an ad hoc task in which participants were required to observe a movement in a video and then to predict the end of the same movement (39, 40, 80). The video was darkened for a given time interval a few seconds after it started so that the task could be performed exclusively by extrapolating the time-related features of the motion sequence being observed. Both patients with task-specific (writer's cramp) (39) or non-task-specific (cervical) dystonia (40) less accurately predicted the temporal outcome of the visually perceived movement of the human body though not of that of an inanimate object. In another study, we showed that the cerebellum is largely involved when temporal information is handled to predict the temporal outcome of a motor act (80). When lateral cerebellum activity was inhibited by using 1 Hz-repetitive transcranial magnetic stimulation, the time required to estimate a movement increased when a body segment was involved, though not when an inanimate object was involved (80). These results are in keeping with those by Filip and co-workers, who showed that predictive motor timing in cervical dystonia patients was impaired when the latter were required to mediate the interception of a moving target (81); this finding was accompanied by cerebellar hypo-activity as well as by connectivity with the basal ganglia and the motor cortex (82). All these deficits may be linked to an abnormal internal model of motor commands that is mainly due to a dysfunction in the cerebellum that prevents the latter from integrating proprioceptive inputs (41). In keeping with this hypothesis, we recently demonstrated that anticipatory movement control is impaired in patients with cervical dystonia (79). The term “anticipatory” indicates the feed-forward portion of a movement that is planned in advance and relies on the internal model of a motor act. Interestingly, we found that abnormal anticipatory control only occurred in a subgroup of cervical dystonia patients with tremor in the dystonic or non-dystonic body parts. This finding suggests that the cerebellum might play a specific role in the occurrence of dystonic tremor, though further studies are needed to confirm this hypothesis.

Illusory perception paradigms activating proprioceptive mechanisms have been used in PD and dystonia to advance our understanding of the pathophysiology of these movement disorders. The RHI paradigm has recently been applied to patients with PD (10) (Table 1). Interestingly, PD patients displayed more proprioceptive drift, both after synchronous (illusion-inducing condition) and asynchronous stroking (control condition), than healthy controls (10). Temporal deficits and proprioceptive impairments may underlie the peculiar pattern of results of PD patients who underwent the RHI paradigm. As hypothesized by the authors, PD patients might have relied more on the remembered visual cues and less on the proprioceptive cues than controls (10). Consequently, the visual capture evoked by the rubber hand itself (83) might have induced a stronger proprioceptive drift. This explanation is in line with evidence showing that PD patients can compensate for proprioceptive deficits by increasing dependence on vision (36, 84). The enhanced illusion in PD may also indicate a weakened sense of the own body, that could in turn facilitate the incorporation of an artificial body part, as observed in specific clinical conditions (i.e., spinal cord injury) (85, 86). Dopaminergic treatment induced patients to be generally more suggestible, but not specifically to the RHI, i.e., patients in the ON-medication state reported more agreement than in the OFF-medication state not only to the illusion-related statement but also to the control statements, whereas proprioceptive drift was not affected by the dopaminergic drugs (10). This suggests that any potential effect of dopamine on sensory deficits in PD is not sufficient to affect the RHI in any specific way.

The application of the RHI paradigm to patients suffering from focal hand dystonia revealed a reduction in proprioceptive drift (9). Conversely, the proprioceptive drift observed in patients with cervical dystonia was similar to that observed in healthy subjects (9). The selective impairment of proprioceptive drift in focal hand dystonia suggests that the synchronous visual-tactile input fails to integrate with the proprioceptive location sense owing to an underlying kinesthetic deficit (9). In this regard, activity in the inferior parietal lobule (22, 87) and cerebellum (22) seems to be implicated in the recalibration of the perceived position of one's own limb and, interestingly, abnormalities in these two regions have been described in different forms of dystonia (88–92). It has hence been suggested that dysfunctions in a circuit involving these two structures may underpin the reduction in proprioceptive drift observed in focal hand dystonia patients (9), which is in keeping with the notion that dystonia is not only related to dysfunctions in the basal ganglia. The use of the RHI in movement disorders may shed light on subtle proprioceptive dysfunctions hypothesized to be involved not only in motor control (as classically considered) but also in higher order functions, such as the sense of the body (13). The different pattern at the RHI in PD and focal-hand dystonia may suggest that different neural circuits involved in body processing are abnormal in the two conditions. Future neuroimaging studies could help to clarify this aspect. Moreover, it is tempting to speculate that the enhancement of the proprioceptive drift in PD and its reduction in focal-hand dystonia could hint at a different strength of the own body representation in the two clinical conditions. As suggested above, a weakened sense of the own body could facilitate incorporation of an artificial limb (86), and this could be the case in PD. Conversely, in focal-hand dystonia the lack of proprioceptive drift on the affected hand could indicate a resistance to the illusion that could be related to a strong sense of the impaired body district. These hypotheses, however, should be proved in *ad-hoc* experimental studies.

## Experimental approaches to investigate actual and illusory tactile information in humans

One of the approaches used most widely to measure the accuracy of temporal processing of sensory information is the somatosensory temporal discrimination threshold (STDT). The STDT is defined as the shortest interval at which the subject identifies two tactile stimuli delivered to the same body part as temporally separate (42). The STDT involves the activation of several cortico-subcortical brain areas (42). Studies have shown that the primary somatosensory cortex encodes and refines the STDT. In healthy subjects, S1 rTMS, a technique that induces changes in cortical activity, modifies STDT values (93) and rTMS-induced changes in STDT values correlate with SEP recovery cycles and S1-high frequency oscillations (HFO), both of which reflect cortical inhibitory interneuron activity (93–97). Moreover, a recent study (95) showed that the interval at which an individual recognizes a third stimulus as clearly distinct after a pair of stimuli delivered at the STDT is shorter than the interval for the STDT, therefore suggesting that the somatosensory system dynamically modulates its activity depending on the time properties of the environmental stimulation (95).

The role played by the basal ganglia in STDT processing has been known for decades. Evidence from animal studies has shown that the basal ganglia filter incoming information. The STDT also impinges on an alerting circuit that signals the detection of biologically salient events through thalamic connections with the basal ganglia (i.e., midbrain dopaminergic neurons) (98). Accordingly, perceptual certainty in a temporal discrimination task is associated with putamen activation (99–101). Subcortical processing in the basal ganglia may therefore signal salient events whereas stimulus-driven rapid plasticity mechanisms, which are mediated by inhibitory interneuron activity, may regulate somatosensory temporal encoding activity in S1 (7).

The so-called Aristotle illusion is an illusory paradigm that has been used to perform a fine-tuned investigation of the somatosensory inter-digits relationships in movement disorders. In this illusion, one object is perceived as two if it is placed in the contact point of crossed fingertips in a blindfolded subject (102). This illusory doubling only occurs in the crossed fingers position, while the parallel fingers position leads to the realistic perception of a single stimulus (103, 104). The illusory doubling perception arises from the interplay between the proprioceptive (the fingers' unusual configuration) and the tactile (the contact of the object on the skin) information, i.e., when the fingers are crossed, the tactile signals are detected by two normally distant skin regions that are usually touched by two objects. Since the brain needs time to readapt the frame of body reference to the new finger configuration, the sensory signals from the fingertip contact point are, in the meantime, processed as if the crossed fingers were in a parallel position and, consequently, two objects are perceived instead of one (105).

## Evidence on actual and illusory tactile information processing in PD and dystonia

In keeping with the hypothesis of a prominent role of dopaminergic neurons in the STDT, several studies have reported increased STDT values in PD (1, 6, 8, 42, 44, 48) (Table 1). STDT abnormalities in PD correlate with the degree of nigrostriatal dopamine loss as well as with the severity and duration of the disease (6, 8, 49); moreover, such abnormalities, improve after dopaminergic medication (42, 44, 48). Since the STDT in PD is normal at disease onset, which suggests that STDT abnormalities only appear when dopamine loss reaches a threshold that may be higher than that associated with the onset of motor symptoms (6), it may be considered as a marker of disease progression (6). Recent studies on PD (45, 50) have suggested that an abnormal STDT might be involved in the development of parkinsonian motor deficits. The correlation between STDT values and variables that assess movement performance point to a possible link between altered STDT and motor disturbances in PD (8, 45). In a study designed to investigate how STDT-related circuits interact with motor performance (50, 106), we observed that the STDT increases at movement onset and returns to baseline values after 200 ms. We suggested that the increase in STDT during movement execution is likely to reflect mechanisms of sensory gating of tactile information that are irrelevant to the ongoing movement. In PD patients, temporal coupling of tactile sensory information and motor outflow is altered (i.e., the STDT increases to a lesser extent during movement and returns to baseline values more quickly than in healthy subjects), thus suggesting that irrelevant incoming information may affect movement. This hypothesis is supported by the observation that STDT testing during index finger abductions in healthy subjects induces no changes in movement kinematics, whereas in PD patients it reduces the mean velocity of finger abductions. STDT alterations in PD thus appear to contribute to motor symptoms via impaired sensorimotor integration.

In dystonia, the STDT is abnormally increased in different forms of focal dystonia in both affected and unaffected body parts. STDT is also impaired in unaffected relatives of dystonic patients (51, 52, 56, 59, 60, 107, 108) (Table 1). Overall, STDT alterations in dystonia seem to represent an endophenotypic feature of the disease. How and where STDT abnormalities play a pathophysiological role in dystonia is an important question that deserves clarification. Do STDT alterations stand as a dystonia endophenotype with no direct effects on motor dysfunctions? Or do STDT abnormalities concur in a second pathophysiological mechanism (i.e., altered sensorimotor integration) to a different extent across the various focal dystonias? The dystonia endophenotype hypothesis is supported by the fact that STDT changes are present when dystonic features are not yet manifest in patients with increased blinking (a prodromal form of blepharospasm) (46, 53) as well as in non-manifesting family members of genetic forms of dystonia (52, 54, 55, 108); moreover, STDT changes in dystonic patients are not modified by botulinum toxin injections (57) or DBS stimulation (61); lastly, in a recent 8-year follow-up study on dystonic patients, we found that STDT values do not change over time despite the progression in dystonia severity, which implies that STDT abnormalities in dystonia may be considered as a “fingerprint” that remains stable over time (109). However, several lines of evidence also point to a possible role of altered temporal processing of tactile information as a factor that concurs with maladaptive cortical plasticity and abnormal sensorimotor integration. When they investigated the neurophysiological correlates of abnormal somatosensory temporal discrimination in dystonia, Antelmi et al. (62) found that increased STDT values were associated with reduced suppression of cortical and subcortical paired-pulse somatosensory evoked potentials as well as with a smaller area of the high-frequency oscillation early component, which points to inefficient inhibitory activity in S1. Since cortical plasticity mechanisms rely on a dynamic balance between excitatory and inhibitory interneurons, altered inhibitory interneuron activity may concur to give rise to other pathophysiological mechanisms in dystonia, such as aberrant cortical plasticity mechanisms (110). Future studies on the relationship between the STDT and movement may help to clarify whether dystonic patients in whom an increased STDT is an endophenotypic feature may also be affected by altered subcortical mechanisms of sensorimotor integration (i.e., gating of tactile stimuli during movement).

In conclusion, STDT abnormalities in dystonia may reflect defective inhibitory activity at both the cortical (S1-meaning a defective refining system) and subcortical levels and create a permissive background for the development of other pathophysiological processes in dystonia. Which of these pathophysiological processes are directly linked to motor symptoms remains unknown.

When the Aristotle illusion paradigm was applied to PD patients, patients and healthy controls were found to experience the same illusory doubling perception (64). Hence, tactile perception involving an inter-digit functional relationship appears to be preserved in PD (64) (Table 1).

Interestingly, the same paradigm applied to patients with dystonia revealed a different pattern of results. The illusion was found not to occur in focal hand dystonia when the non-affected fingers of the affected hand were touched, whereas it did occur when the object came into contact with the affected fingers (65). This study thus demonstrated a very specific tactile alteration that was present in the affected hand of patients with focal hand dystonia though not in patients with other types of dystonia, such as cervical dystonia and blepharospasm (65). This finding suggests that the impairment in inter-digit tactile perception is, unlike other kinds of tactile deficits, specific to focal hand dystonia.

Since the Aristotle illusion is associated above all with primary somatosensory cortex activity (105), the fact that this illusion is selectively altered in focal hand dystonia (65) highlights the importance of the role played by somatosensory cortical alterations in this form of dystonia though not in other types of dystonia, such as blepharospasm and cervical dystonia (65), or in PD (64). Following the same line of reasoning, the reduction in Aristotle's illusion in focal hand dystonia may be interpreted as the behavioral consequence of alterations in the extent of cortical activation induced by tactile stimulation (65). This hypothesis is supported by the fact that finger representation in the primary somatosensory cortex is abnormal in focal hand dystonia (111–113). By contrast, it has been suggested that the functional activation of finger representation in the somatosensory cortex is retained in patients with PD, which may explain why the functional relationship between fingers during tactile perception is preserved in this disease (64). Moreover, the results of a neurophysiological study (105) suggest that the mechanisms underlying Aristotle's illusion are related above all to activity in the primary somatosensory cortex rather than in the basal ganglia.

## General summary and hypotheses for potential therapeutic strategies

In this narrative review we have described a number of abnormalities in somatic sensory input processing that have been reported in PD and dystonia. We have also shown that these abnormalities are not limited to unimodal sensory processing but are also very evident in somatosensory (when different sensory modalities are integrated, as in the case of haptic perception or illusion) and sensorimotor integration processes.

The cerebral network underlying these abnormal processes of integration is likely to include the basal ganglia (as a key node) and the sensorimotor areas of the cerebral cortex for PD, and to extend to the cerebellum in dystonia. Although the extent to which somatic sensory processing and integration mechanisms contribute to the mechanisms underlying motor disturbances in PD and dystonia has only partially been investigated to date, the possibility of manipulating sensory information to improve motor deficits in PD and dystonia deserves further attention.

Sensorimotor retraining may serve as a therapeutic strategy in movement disorders. There is some evidence that the modulation of somatosensory cortical activity by means of non-invasive brain stimulation improves motor symptoms and induces self-perceived improvement (114–116). Furthermore, in patients with PD as well as in patients with focal dystonia muscle vibration, transcutaneous electrical nerve stimulation and kinesio-taping used for sensory retraining purposes seems to improve sensory and motor disturbances (117–122).

In this vein of thought and, more importantly, by considering the integrity of the sensory pathways in PD and dystonia, the active retraining of proprioceptive and tactile discriminative processing may prove to be even more effective than a short-lasting external modulation of sensory areas or a passive engagement of sensory afferent pathways. The objective of such training would be to improve spatial and temporal processing capacities through guided activities that may enhance different aspects of sensory feedback, including somatosensation, proprioception, and kinesthesia, thus ameliorating sensorimotor integration, reducing dependence on visual inputs and possibly restoring skills in patients with movement disorders.

If taken together, the observations derived from the rubber hand illusion and the Aristotle illusion suggest that these paradigms may help to shed new light on the mechanisms underlying specific sensory alterations in different types of movement disorders. Some of the alteration patterns uncovered by these illusions so far appear to be distinctive features of focal hand dystonia though not of other movement disorders. Specifically, it appears that the deficits emerging in focal-hand dystonia (i.e., lack of proprioceptive drift at the RHI and reduced Aristotle illusion) are specific to the affected body part. Moreover, while in focal-hand dystonia there is a resistance to the illusions, in PD there seems to be a facilitation. Whether the localization of motor symptoms to one body district in focal-hand dystonia attracts patients' attention or increase the sense of the own body part so as to reduce the illusory sensation in this condition and whether a more malleable body representation in PD patients facilitates the illusions should be proved in *ad-hoc* studies.

In PD, the feasibility and efficacy of exploiting illusion for a rehabilitative purpose has been demonstrated using the Mirror visual feedback (MVF) therapy (123). When combined with motor training, MVF has been proven to improve the performance of the trained and untrained hand by enhancing the excitability of both primary motor cortices in healthy subjects (124, 125). In a pilot study, it has been shown that a unilateral hand training performed by PD patients with the less affected side and based on MVF was able to induce changes in bradykinesia of the untrained (and more affected) hand (123).

Bearing in mind the abnormalities associated with illusions of sensory information in patients with PD and dystonia, therapeutic retraining may also include approaches using bodily illusions. Indeed, paradigms based on bodily illusions have been exploited for rehabilitative purposes in numerous clinical contexts (126). For instance, the RHI has been applied to induce the re-attribution of the hand in somatoparaphrenia (127), to improve left spatial neglect in right-brain damaged patients (128), to alleviate pain in complex regional pain syndrome (129), and cervical spinal cord injury (130) as well as to help to incorporate the prosthesis in amputees (131–133). These examples suggest that multisensory stimulation related to body awareness may induce beneficial effects and might inspire the development of new rehabilitation strategies even for movement disorders. A potential avenue for research could be also to use bodily illusions to alleviate pain that could be present as non-motor symptom in patients with Parkinson's disease.

## Author contributions

AC conceptualized the work; LA, MF, and AC wrote the first draft; AC revised the draft.

### Conflict of interest statement

The authors declare that the research was conducted in the absence of any commercial or financial relationships that could be construed as a potential conflict of interest. The reviewer MP declared a shared affiliation, with no collaboration, with one of the authors, AC, to the handling editor at time of review.
